# Correction: A new adenine nucleotide transporter located in the ER is essential for maintaining the growth of *Toxoplasma gondii*

**DOI:** 10.1371/journal.ppat.1010998

**Published:** 2022-11-29

**Authors:** Senyang Li, Jiahui Qian, Ming Xu, Jing Yang, Zhengming He, Tongjie Zhao, Junlong Zhao, Rui Fang

The gene ID of TgANT appears incorrectly. The correct gene ID is TGGT1_254580.

In the TgANT is an ER membrane protein in *T*. *gondii* subsection of the Results, there are errors in the last three sentences of the first paragraph. The correct sentences are: Among the sixty-nine selected genes, we focused on *TGGT1_254580*, which was annotated as UDP-galactose transporter family protein in the *ToxoDB* database, and predicted to have ten transmembrane helices (Fig 1A and 1B). Besides, *TGGT1_254450* contains a C-terminal dilysine motif (-KKQC) that serves as an ER retention signal, C-terminally dilysine motif shown in other proteins to limit exit from the ER (Fig 1A) [21]. According to the nomenclature in other species, we named *TGGT1_254450* as *T*. *gondii* Adenine Nucleotide Transporter (TgANT), and distinguished from mitochondrial ATP/ADP carrier (AACs).

## References

[1] Li S, Qian J, Xu M, Yang J, He Z, Zhao T, et al. (2022) A new adenine nucleotide transporter located in the ER is essential for maintaining the growth of *Toxoplasma gondii*. PLoS Pathog 18(7): e1010665. doi: 10.1371/journal.ppat.1010665 35788770 PMC9286291

