# Supplementary figures and images for: A new adenine nucleotide transporter located in the ER is essential for maintaining the growth of Toxoplasma gondii
Source: PLoS Pathog. 2022 Jul 5;18(7):e1010665. doi: 10.1371/journal.ppat.1010665 (PMC9286291; doi:10.1371/journal.ppat.1010665)

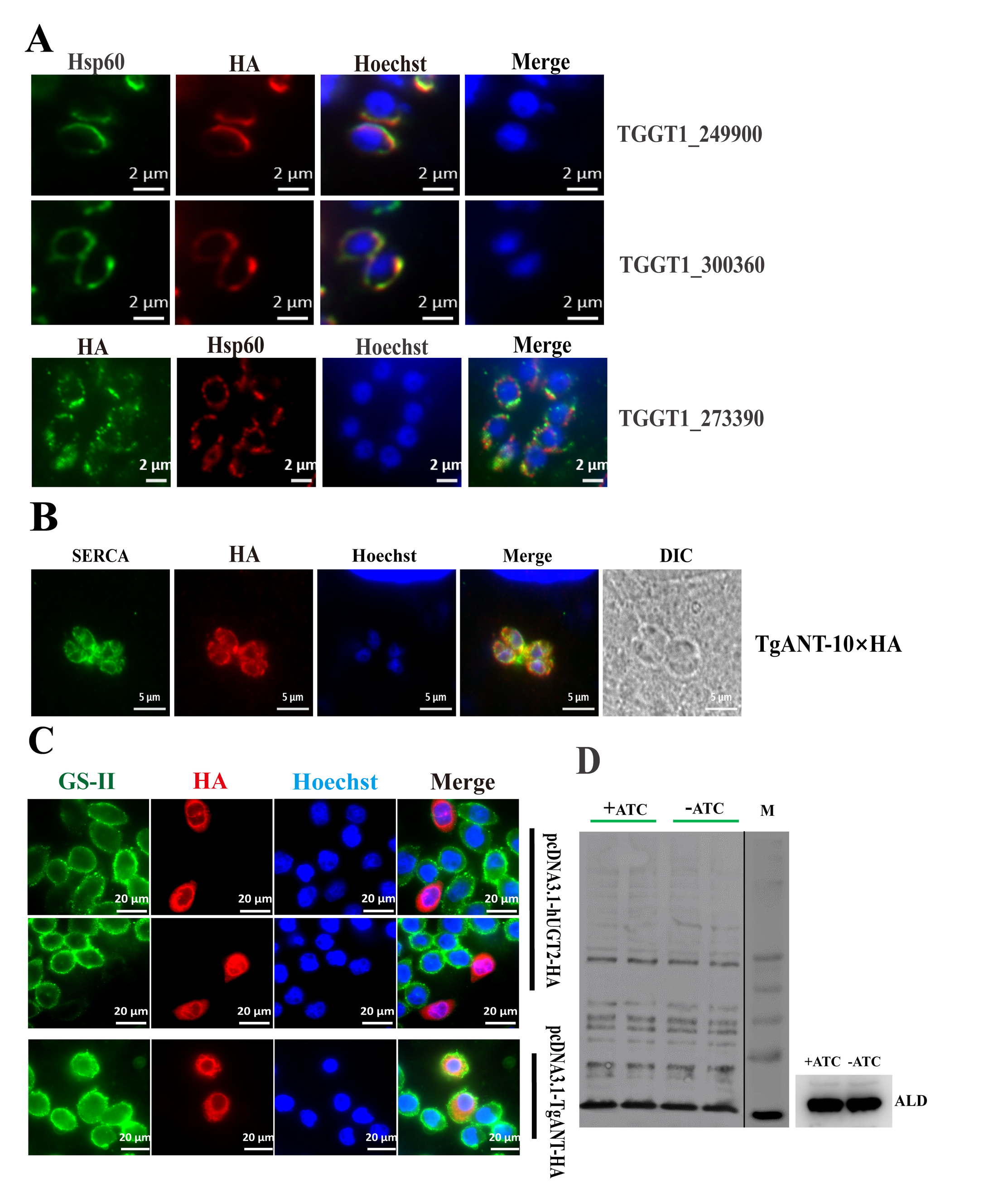

Supplement: S1 Fig — (A) To determine the subcellular location of TGGT1_273390, TGGT1_300360 and TGGT1_249900 in T.gondii, 3×HA-tagged was cloned into the C-terminus by CRISPR-Cas9–mediated site-specific integration in the RHΔku80 strain. IFA confirmed the subcellular location of TGGT1_273390, TGGT1_300360, TGGT1_249900. Hsp60(Heat shock protein 60) as a marker of mitochondrial. Scale bars: 2 μm. (B) IFA staining to determine the subcellular location of TgANT-10×HA strain.(C) Lec8 cells were transfected with pcDNA3.1- TgANT-HA or with pcDNA3.1- hUGT2-HA, followed cells were examined for the binding of GS-II-488 (green) and anti-HA monoclonal antibody(red). Bar, 20μm. (D) The iTgANT strain was treated with 0.5 μg/ml ATc or left untreated, then pipetted with the syringe and filtered to collect the tachyzoite. Lectin GS-II-488 was used to detect the galactosylation level of these two samples by lectin blot. (TIF) [file ppat.1010665.s001.tif]

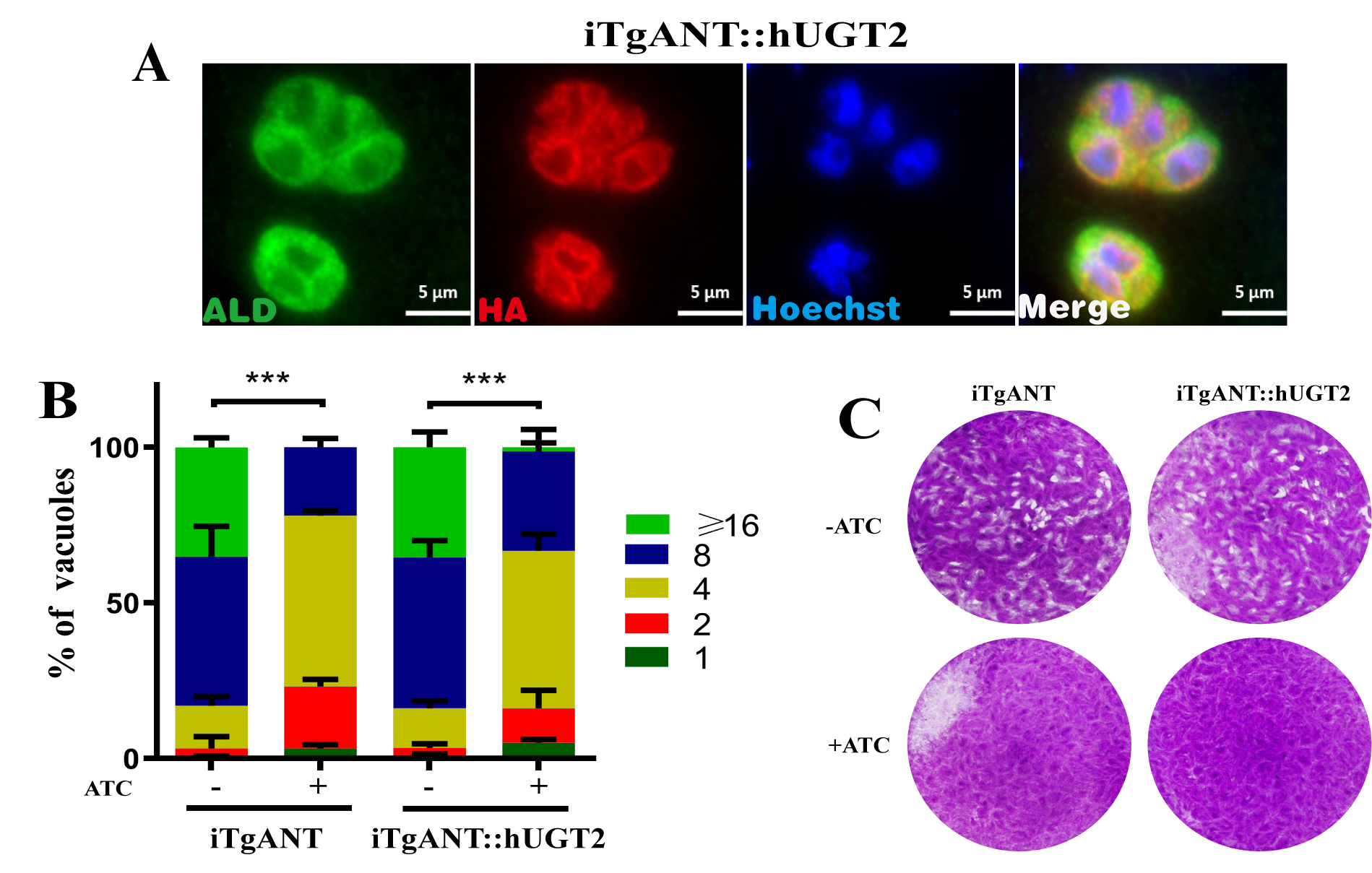

Supplement: S2 Fig — (A) IFA confirmed the correct integration and expression in ER of iTgANT::hUGT2 strain. (B) and (C) Intracellular replication assay(B) and plaque assay(C) comparing the growth of TgANT depletion strain and hUGT2 complementation strain. Means ± s.e.m. ***p ≤ 0.001, two-way ANOVA, three independent repeated, a representative one is shown here. (TIF) [file ppat.1010665.s002.tif]
