# Supplementary material for: A new adenine nucleotide transporter located in the ER is essential for maintaining the growth of Toxoplasma gondii
Source: PLoS Pathog. 2022 Jul 5;18(7):e1010665. doi: 10.1371/journal.ppat.1010665 (PMC9286291; doi:10.1371/journal.ppat.1010665)
Supplement: S2 Table — (DOCX) [file ppat.1010665.s004.docx]

| S4-1 Table. Plasmids used in this study | | |
| --- | --- | --- |
| Name of plasmids | **Use** | **Construction methods or** **source** |
| pSAG1-Cas9-sgUPRT | Template for gene specific CRISPR plasmid construction and the UPRT specific CRISPR plasmid | [1] |
| pSAG1-Cas9-sgTgANT-cKO | *TgANT* specific CRISPR plasmid for the iPYK1 construction | Site-directed mutagenesis to replace the gRNA in pSAG1-Cas9-sgUPRT with gene specific gRNAs. |
| p7TetOS1 | Template for SAG1-TetO7 promoter amplification | [2] |
| pTub:: TgANT::HA:: *CAT* | To construct the TgANT comp strain | The coding sequence of TgANT was cloned into pPYK1::DHFR [2] |
| pTub:: hUGT2::HA:: *CAT* | Express:: hUGT2 in the iTgANT strain | The coding sequence of hUGT2 was cloned into pPYK1::DHFR [2] |
| pTub:: AXER::HA:: *CAT* | Express AXER in the iTgANT strain | The coding sequence of AXER was cloned into pPYK1::DHFR |
| pTub:: TgANT(^335^SSxQQ) ::HA::*CAT* | Express mutational TgANT in the iTgANT strain | The coding sequence of TgANT **(**^335^SSxQQ**)** was cloned into pPYK1::DHFR [2] |
| pTub:: TgANT(^173^YsxxxxQxxQ ) ::HA:: *CAT* | Express mutational TgANT in the iTgANT strain | The coding sequence of TgANT(^173^YsxxxxQxxQ ) was cloned into pPYK1::DHFR |
| pET16b | To construct the prokaryotic expression vector of TgANT or mutational TgANT | This work |
| pUC19 | Template for pUC19 amplification | From the Sibley Lab |
| pCDNA3.1 | To construction of eukaryotic expression vector of TgANT or hUGT2 | This work |

**S4-2 Table. Transgenic parasites used in this study**

| Strain | Description | Source |
| --- | --- | --- |
| TATi | Parental strain | [3] |
| iTgANT | TgANT conditional knockdown | This work |
| RH *Δhxgprt* | Parental strain | From the Sibley Lab |
| Ku80 | Parental strain | From the Sibley Lab |
| iTgANT::AXER | Heterologous complementation strains AXER -HA expressed in iTgANT | This work |
| iTgANT::hUGT2 | Heterologous complementation strains hUGT2-HA expressed in iTgANT | This work |
| Com-TgANT-^335^SSxQQ | Mutational TgANT expressed in iTgANT | This work |
| Com-TgANT-^173^YsxxxxQxxQ | Mutational TgANT expressed in iTgANT | This work |

**References:**

1. Shen B, Brown KM, Lee TD, Sibley LD. Efficient gene disruption in diverse strains of *Toxoplasma gondii* using CRISPR/CAS9. mBio 5, e01114-01114 (2014).
2. Xia N, Ye S, Liang X, Chen P, Zhou Y, Fang R, Zhao J, Gupta N, Yang S, Yuan J, Shen B. Pyruvate Homeostasis as a Determinant of Parasite Growth and Metabolic Plasticity in *Toxoplasma gondii*. mBio. 10(3): e00898-19(2019).
3. Meissner M, Schluter D, Soldati D. Role of *Toxoplasma gondii* myosin A in powering parasite gliding and host cell invasion. Science 298, 837-840 (2002).
