# Supplementary material for: A new adenine nucleotide transporter located in the ER is essential for maintaining the growth of Toxoplasma gondii
Source: PLoS Pathog. 2022 Jul 5;18(7):e1010665. doi: 10.1371/journal.ppat.1010665 (PMC9286291; doi:10.1371/journal.ppat.1010665)
Supplement: S4 Table — (DOC) [file ppat.1010665.s006.doc]

The amino acid sequences of AXER fusion protein: the green part is the first 63 amino acid sequences of TgANT, the gray part is the amino acid sequence of AXER, and the red part is the HA epitope.

**MAAGSAMVDKGVSPVARRKDKHVTGAGPSSPFSNLSTTSGQDEAQSKAGSPVAQEKATGVDSTNASSSSLVPDRLRLPLCFLGVFVCYFYYGILQEKITRGKYGEGAKQETFTFALTLVFIQCVINAVFAKILIQFFDTARVDRTRSWLYAACSISYLGAMVSSNSALQFVNYPTQVLGKSCKPIPVMLLGVTLLKKKYPLAKYLCVLLIVAGVALFMYKPKKVVGIEEHTVGYGELLLLLSLTLDGLTGVSQDHMRAHYQTGSNHMMLNINLWSTLLLGMGILFTGELWEFLSFAERYPAIIYNILLFGLTSALGQSFIFMTVVYFGPLTCSIITTTRKFFTILASVILFANPISPMQWVGTVLVFLGLGLDAKFGKGAKKTSHYPYDVPDYA**

The nucleoside sequences of AXER fusion protein: the green part is the first 63 nucleoside sequences of TgANT, the gray part is the nucleoside sequences of AXER, and the red part is the HA epitope.

atgGCCGCAGGGTCCGCTATGGTGGACAAGGGCGTTTCGCCGGTTGCTCGTCGGAAAGACAAACATGTGACCGGCGCGGGGCCCTCCTCCCCGTTTAGCAACCTTTCCACGACCTCCGGGCAGGATGAGGCCCAGAGCAAGGCTGGTTCGCCTGTCGCCCAAGAGAAGGCGACCGGCGTGGACAGCACCAACGCCTCTAGCAGCTCCCTGGTGCCCGACCGGCTGCGCCTGCCGCTCTGCTTCCTGGGTGTCTTTGTCTGCTATTTTTACTATGGGATCCTGCAGGAAAAGATAACAAGAGGAAAGTATGGGGAAGGAGCCAAGCAGGAGACGTTCACCTTTGCCTTAACTTTGGTCTTCATTCAATGTGTGATCAATGCTGTGTTTGCCAAGATCTTGATCCAGTTTTTTGACACTGCCAGGGTGGATCGTACCCGGAGCTGGCTCTATGCTGCCTGTTCTATCTCCTATCTGGGTGCCATGGTCTCCAGCAATTCAGCACTACAGTTTGTCAACTACCCAACTCAGGTCCTTGGTAAATCCTGCAAGCCAATCCCAGTCATGCTCCTTGGGGTGACCCTCTTGAAGAAGAAGTACCCGTTGGCCAAGTACCTGTGTGTGCTGTTAATTGTGGCTGGAGTGGCCCTTTTCATGTACAAACCCAAGAAAGTTGTTGGGATAGAAGAACACACAGTCGGCTATGGAGAGCTACTCTTGCTATTATCGCTGACCCTGGATGGACTGACTGGTGTTTCCCAGGACCACATGCGGGCTCATTACCAAACAGGCTCCAACCACATGATGCTGAACATCAACCTTTGGTCGACATTGCTGCTGGGAATGGGAATCCTGTTCACTGGGGAGCTCTGGGAGTTCTTGAGCTTTGCTGAAAGGTACCCTGCCATCATCTATAACATCCTGCTCTTTGGGCTGACCAGTGCCCTGGGTCAGAGCTTCATCTTTATGACGGTTGTGTATTTTGGTCCCCTGACCTGCTCCATCATCACTACAACTCGAAAGTTCTTCACAATTTTGGCCTCTGTGATCCTCTTCGCCAATCCCATCAGCCCCATGCAGTGGGTGGGCACTGTGCTTGTGTTCCTGGGTCTTGGTCTTGATGCCAAGTTTGGGAAAGGAGCTAAGAAGACATCCCACtatccttacgatgttccagattatgccTGA
