# Supplementary material for: A new adenine nucleotide transporter located in the ER is essential for maintaining the growth of Toxoplasma gondii
Source: PLoS Pathog. 2022 Jul 5;18(7):e1010665. doi: 10.1371/journal.ppat.1010665 (PMC9286291; doi:10.1371/journal.ppat.1010665)
Supplement: S5 Table — (DOC) [file ppat.1010665.s007.doc]

**Optimization Report**

**Sequence optimization information:**

Expression System:*Escherichia coli*

Gene Length:1242 (bp)

**1.Codon Used Adjustment**

The best value is 1 for sequence optimization.


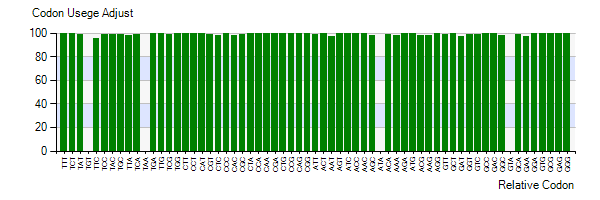


CAI: 0.71

Before Codon Adjustment


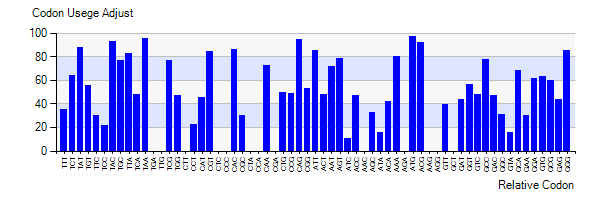


CAI: 0.90

After Codon Adjustment

**2.Codon Used Distribution**

Show the relative codon used distribution


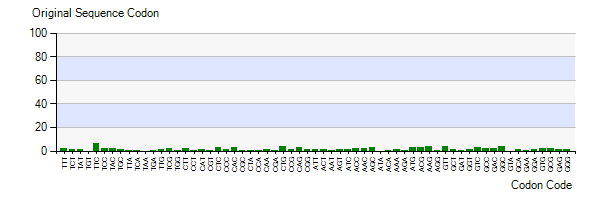


Before Optimization


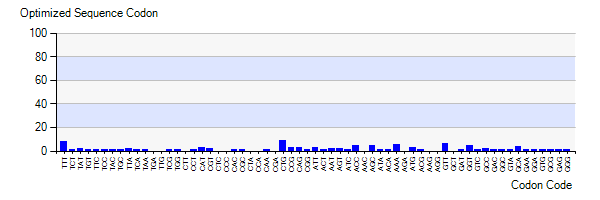


After Optimization

**3.GC Content:**

The comparison of GC content between original sequence and optimized sequence


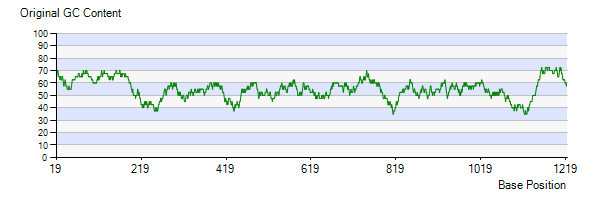


Before Optimization


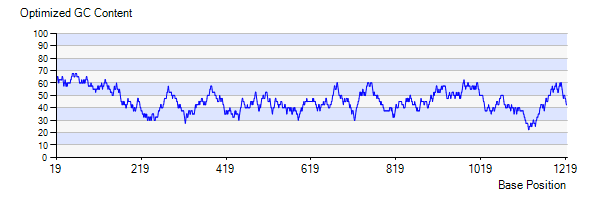


After Optimization

**4.Restriction Enzyme**

| Restriction Name | Original | Optimization |
| --- | --- | --- |
| BamHI | 0 | 0 |
| XhoI | 0 | 0 |

**5.Removed Repeats**

Before Optimization

After Optimization

Max Direct Length:14

**6.Optimized Sequence**

ATGGCAGCCGGGAGCGCAATGGTTGACAAAGGAGTGAGCCCGGTTGCGCGCCGCAAAGACAAACACGTTACGGGGGCGGGTCCGTCGTCGCCTTTTTCGAATCTGAGCACGACAAGCGGGCAGGATGAGGCACAAAGCAAAGCAGGAAGCCCGGTTGCACAGGAAAAAGCAACCGGAGTCGATAGTACAAATGTTCGTCAAGTTCTGCTGATGGTTGTGATTGTTACCGGCATTTATTTTTTTTTCCTGATGTTTGGTTACTACCAGGAACAGATATACCATCAGGTAGACCCGACCACCGGCAAACGTTTTTCATTTAGTTTTTTTCTGGTGTTCGTGATTTGCGCAAGCAATTCTTTTTTTAGCCTGGGTCTGCTGCTGTTACAGACACGTGGCAATGCACAGAAAGCCTTTCATGTTCTGGATACCTTTATTTTTCGTGAGGTTCTGTTTATTTCCCTGAGTTATAGCGGTGCCATGTTATGTACGAATTATGCCCTGACCCACGTTAATTATCCGACCCAGGTTCTGGTTAAATCTGCAAAAATGGTTCCGATCGTTCTGGGTGGTTTTTTTGTTTTTCGTAAAACGTATCCGTGGTATGACTATCTGAGTGTTGCAGTCGTTACTGTTAGCCTGGTTCTGTTTAATTTTGCCAAAGCGGGTAGTAGCAGCAAACATACGGAGAGCACCGCAGTCGGTATTTTACTGTTATGTGTTAGCCTGTTTTGTGATGGTTTAACCGGACCGCGGCAAGATCGTCTGATGGCCCGTTACACTAATTTAGGTCCGGTGTTAATGATGTTTCTGACCAATCTGTTTTCTACTGTTTGGACCGCAATTGCGAGTCTGTTAATTGAGGGTGAACAACCGTTTCTGTTTCTGAAACATGATCCGGATGCGTTAGGTAGTTTTGCCGCGTTTACCGTGAGCGGTTCTCTGGGTCAGCTGTTTATTTACCAGTCACTGCGGGCATTTGGGAGCCTGTACACCAGCCTGTTTACCACCCTGCGTAAAGCCACGAGCACCGTTTTAAGTGTTTATATTTTTGGGCATCACATGACCCCGGTTCAGTGGATTAGCATGTTTTGTATTTTTCTGACCCTGCTGGTTCAAAGCTATTGCTCAAAAAAATTTAAAAAACATGGTAAAGGTCATCGTCATCATGGTCATCATGGTCATAAAGAACCGGCAAGCCCGACCGCACATGGTGGTGAAAATGCACTGAAAAAACAGTGTTAA

**7.Protein Alignment**

Original Protein

MAAGSAMVDKGVSPVARRKDKHVTGAGPSSPFSNLSTTSGQDEAQSKAGSPVAQEKATGVDSTNVRQVLLMVVIVTGIYFFFLMFGYYQEQIYHQVDPTTGKRFSFSFFLVFVICASNSFFSLGLLLLQTRGNAQKAFHVLDTFIFREVLFISLSYSGAMLCTNYALTHVNYPTQVLVKSAKMVPIVLGGFFVFRKTYPWYDYLSVAVVTVSLVLFNFAKAGSSSKHTESTAVGILLLCVSLFCDGLTGPRQDRLMARYTNLGPVLMMFLTNLFSTVWTAIASLLIEGEQPFLFLKHDPDALGSFAAFTVSGSLGQLFIYQSLRAFGSLYTSLFTTLRKATSTVLSVYIFGHHMTPVQWISMFCIFLTLLVQSYCSKKFKKHGKGHRHHGHHGHKEPASPTAHGGENALKKQC*

Optimized Protein

MAAGSAMVDKGVSPVARRKDKHVTGAGPSSPFSNLSTTSGQDEAQSKAGSPVAQEKATGVDSTNVRQVLLMVVIVTGIYFFFLMFGYYQEQIYHQVDPTTGKRFSFSFFLVFVICASNSFFSLGLLLLQTRGNAQKAFHVLDTFIFREVLFISLSYSGAMLCTNYALTHVNYPTQVLVKSAKMVPIVLGGFFVFRKTYPWYDYLSVAVVTVSLVLFNFAKAGSSSKHTESTAVGILLLCVSLFCDGLTGPRQDRLMARYTNLGPVLMMFLTNLFSTVWTAIASLLIEGEQPFLFLKHDPDALGSFAAFTVSGSLGQLFIYQSLRAFGSLYTSLFTTLRKATSTVLSVYIFGHHMTPVQWISMFCIFLTLLVQSYCSKKFKKHGKGHRHHGHHGHKEPASPTAHGGENALKKQC*

**8.DNA Alignment**

Original DNA

ATGGCCGCAGGGTCCGCTATGGTGGACAAGGGCGTTTCGCCGGTTGCTCGTCGGAAAGACAAACATGTGACCGGCGCGGGGCCCTCCTCCCCGTTTAGCAACCTTTCCACGACCTCCGGGCAGGATGAGGCCCAGAGCAAGGCTGGTTCGCCTGTCGCCCAAGAGAAGGCGACCGGCGTGGACAGCACCAACGTCCGGCAAGTTCTTCTGATGGTTGTGATTGTCACGGGGATCTACTTTTTCTTCCTCATGTTTGGGTACTATCAGGAACAGATCTACCACCAGGTGGACCCGACTACCGGAAAGCGCTTTTCGTTCTCCTTTTTCCTCGTCTTCGTCATCTGCGCCTCCAACAGCTTCTTCAGTCTTGGCTTGCTGCTTCTGCAGACGCGAGGCAACGCACAAAAGGCCTTCCACGTTCTAGACACGTTCATTTTCAGAGAGGTTCTCTTCATTTCCCTCTCGTACTCGGGCGCAATGCTCTGCACGAACTATGCGCTCACGCATGTCAACTATCCAACGCAAGTCCTCGTGAAGTCTGCGAAAATGGTTCCGATCGTCCTGGGCGGCTTCTTCGTTTTCCGCAAGACGTACCCGTGGTATGACTACCTGTCTGTGGCGGTTGTCACCGTTTCGTTGGTTCTTTTCAACTTCGCGAAGGCAGGAAGCAGCAGCAAACACACCGAGAGCACAGCCGTTGGCATCTTGCTGCTTTGCGTGTCTCTGTTTTGCGACGGGCTCACTGGACCCCGCCAAGATCGGCTGATGGCAAGGTACACGAACCTCGGTCCCGTTCTGATGATGTTTTTGACGAATCTTTTCTCGACAGTTTGGACGGCGATCGCCTCTCTGCTGATTGAAGGCGAACAGCCGTTCCTTTTCCTGAAGCATGACCCAGACGCTCTAGGTAGTTTCGCGGCTTTTACTGTCAGCGGCTCTCTCGGCCAGCTGTTCATTTATCAGTCGCTCCGAGCGTTCGGGAGTCTGTACACCAGCTTGTTCACGACTCTGCGGAAGGCGACCAGCACAGTCCTCAGTGTGTACATTTTCGGACACCACATGACGCCTGTCCAGTGGATCAGCATGTTTTGCATTTTCCTCACTCTCTTAGTTCAGAGCTACTGCTCAAAGAAATTCAAAAAGCATGGCAAGGGCCACCGGCACCACGGCCACCATGGCCACAAAGAGCCGGCCTCCCCCACTGCTCACGGCGGTGAGAATGCCCTGAAGAAGCAGTGCTGA

Optimized DNA

ATGGCAGCCGGGAGCGCAATGGTTGACAAAGGAGTGAGCCCGGTTGCGCGCCGCAAAGACAAACACGTTACGGGGGCGGGTCCGTCGTCGCCTTTTTCGAATCTGAGCACGACAAGCGGGCAGGATGAGGCACAAAGCAAAGCAGGAAGCCCGGTTGCACAGGAAAAAGCAACCGGAGTCGATAGTACAAATGTTCGTCAAGTTCTGCTGATGGTTGTGATTGTTACCGGCATTTATTTTTTTTTCCTGATGTTTGGTTACTACCAGGAACAGATATACCATCAGGTAGACCCGACCACCGGCAAACGTTTTTCATTTAGTTTTTTTCTGGTGTTCGTGATTTGCGCAAGCAATTCTTTTTTTAGCCTGGGTCTGCTGCTGTTACAGACACGTGGCAATGCACAGAAAGCCTTTCATGTTCTGGATACCTTTATTTTTCGTGAGGTTCTGTTTATTTCCCTGAGTTATAGCGGTGCCATGTTATGTACGAATTATGCCCTGACCCACGTTAATTATCCGACCCAGGTTCTGGTTAAATCTGCAAAAATGGTTCCGATCGTTCTGGGTGGTTTTTTTGTTTTTCGTAAAACGTATCCGTGGTATGACTATCTGAGTGTTGCAGTCGTTACTGTTAGCCTGGTTCTGTTTAATTTTGCCAAAGCGGGTAGTAGCAGCAAACATACGGAGAGCACCGCAGTCGGTATTTTACTGTTATGTGTTAGCCTGTTTTGTGATGGTTTAACCGGACCGCGGCAAGATCGTCTGATGGCCCGTTACACTAATTTAGGTCCGGTGTTAATGATGTTTCTGACCAATCTGTTTTCTACTGTTTGGACCGCAATTGCGAGTCTGTTAATTGAGGGTGAACAACCGTTTCTGTTTCTGAAACATGATCCGGATGCGTTAGGTAGTTTTGCCGCGTTTACCGTGAGCGGTTCTCTGGGTCAGCTGTTTATTTACCAGTCACTGCGGGCATTTGGGAGCCTGTACACCAGCCTGTTTACCACCCTGCGTAAAGCCACGAGCACCGTTTTAAGTGTTTATATTTTTGGGCATCACATGACCCCGGTTCAGTGGATTAGCATGTTTTGTATTTTTCTGACCCTGCTGGTTCAAAGCTATTGCTCAAAAAAATTTAAAAAACATGGTAAAGGTCATCGTCATCATGGTCATCATGGTCATAAAGAACCGGCAAGCCCGACCGCACATGGTGGTGAAAATGCACTGAAAAAACAGTGTTAA

**9.Codon Used Table**

TTT [56791] TCT [30494] TAT [50400] TGT [13662]

TTC [32513] TCC [22637] TAC [27239] TGC [12777]

TTA [40627] TCA [30502] TAA [4664] TGA [2674]

TTG [30084] TCG [19071] TAG [751] TGG [31207]

CTT [33816] CCT [22121] CAT [28919] CGT [37134]

CTC [22074] CCC [14379] CAC [17117] CGC [32720]

CTA [12951] CCA [21237] CAA [33607] CGA [11216]

CTG [87261] CCG [33795] CAG [62329] CGG [18434]

ATT [68942] ACT [30518] AAT [68348] AGT [30749]

ATC [45213] ACC [44139] AAC [47233] AGC [33255]

ATA [31065] ACA [35293] AAA [86726] AGA [16583]

ATG [55356] ACG [31794] AAG [35652] AGG [9238]

GTT [50261] GCT [44034] GAT [78663] GGT [55283]

GTC [30515] GCC [50411] GAC [41619] GGC [47962]

GTA [30461] GCA [53619] GAA [81727] GGA [31729]

GTG [46309] GCG [49169] GAG [45154] GGG [28720]
