# Supplementary material for: A new adenine nucleotide transporter located in the ER is essential for maintaining the growth of Toxoplasma gondii
Source: PLoS Pathog. 2022 Jul 5;18(7):e1010665. doi: 10.1371/journal.ppat.1010665 (PMC9286291; doi:10.1371/journal.ppat.1010665)
Supplement: Attachment — Submitted filename: Response to Reviewers.docx [file ppat.1010665.s008.docx]

**Part I - Summary**
Please use this section to discuss strengths/weaknesses of study, novelty/significance, general execution and scholarship.

Reviewer #1: This is a very interesting study, showing that the T. gondii UGT family protein TGGT1_254480 is important for the growth of T. gondii, and examining the consequences of its depletion on ER processes. It provides multiple lines of evidence that, despite its annotation, the protein is not a UDP-galactose transporter. Data consistent with an ER localisation and an ATP/ADP exchange function are presented. The study is novel and significant. Including data for PfUGT, which are eluded to in the text but not shown, would increase its significance further. There are some issues in the manuscript that require clarification and additional details that are needed. Furthermore, the transport studies would benefit from additional controls, and the localisation studies from additional markers.

Reviewer #2: In this manuscript, the authors characterized a Toxoplasma ortholog of ADP/ATP transporter. To determine the substrates of TgANT, the authors cloned this gene in E. coli and identified its capability of importing ATP. In addition, the authors complemented the TgANT KO with human UGT2 and did not observe complementation on growth. However, the complementation with the AXER gene rescued the growth. These assays presented solid evidence that the TgANT transports ATP/ADP and is essential for parasite growth. But, the study lacks mechanistic characterization of how the deletion of TgANT affects ER physiology, further causing parasite death.

Reviewer #3: In this manuscript, the authors describe the identification of the first ER-located protozoan ATP-ADP exchanger in a protozoan species. This is a commendable study, breaking new ground in an important aspect of the cell biology of protozoa, and specifically, apicomplexan parasites. I find the results clearly presented and the conclusions well sustained by the presented evidence, with few exceptions. The manuscript is clearly written and structured and easy to follow, although there are many small issues with written English that need attention, in my opinion.
I have no major comments to make on the scientific validity of this work but do have a number of comments and suggestions for the improvement of the paper. Most of these are minor.

**Part II – Major Issues: Key Experiments Required for Acceptance**
Please use this section to detail the key new experiments or modifications of existing experiments that should be absolutely required to validate study conclusions.

Generally, there should be no more than 3 such required experiments or major modifications for a "Major Revision" recommendation. If more than 3 experiments are necessary to validate the study conclusions, then you are encouraged to recommend "Reject".

**Reviewer #1:** The following additions would strengthen the study, but may not be absolutely required for acceptance:
1. additional controls in the E. coli transport experiments
2. a plasma membrane marker in the localisation studies
3. the data for the function of PfUGT
**These suggestions are outlined in more detail in the next section.**

**Reviewer #2:** 1. Line 124-137, TGGT1_273390, TGGT1_300360, and TGGT1_249900 are highly homologous to ER-ANT1; why are they excluded from being considered as putative TgANT1. At the end of this paragraph, please conclude which gene is named TgANT1 for clarity.

**Response：** We are sorry for the lack of clarity in the description in the manuscript, we have made changes. Since ER-ANT1 has high homology (more than 60%) with the mitochondrial ATP/ADP transporter of Arabidopsis[1], these three proteins in *T. gondii* found by Blastp analysis are also located in mitochondria, rather than located in the ER. At present, the functions of these three proteins are still under study in our laboratory, but the localization results have confirmed that these three proteins are located in mitochondria, not in the ER (Fig. 1). We have summarized in the manuscript which gene is named TgANT.

**Fig1. intracellular localization of** **TgGT1-273390, TgGT1-300360, and TgGT1-249900.**

To determine the subcellular location of TgGT1-273390, TgGT1-300360, and TgGT1-249900 in *T.gondii*, 10×HA-tagged was cloned into the C-terminus by CRISPR-Cas9–mediated site-specific integration in the RHΔku80 strain. Hsp60 is a mitochondrial marker. Scale bars: 2 μm

2. Line 138-140, please explain why the peptide fragment outside transmembrane regions of TgANT1 is not available. Can it not be predicted by topology prediction software?
**Response：** I think our description in the manuscript was not clear enough, which caused confusion for readers. We attempted prokaryotic expression using three peptides outside the transmembrane domain of the TgANT gene, and then prepared polyclonal antibodies. However, the polyclonal antibody we prepared could not detect TgANT protein, either by Western blot or IFA. We have revised this description in our manuscript.

The specific method is as follows:

Three peptides outside the transmembrane domain (8-64, 92-130, 380-407) were amplified by specific primers, and then the three peptides were fused with a prokaryotic expression vector (peSUMO-TgANT) used the homologous recombination method. The IPTG-induced *E. coli* harboring peSUMO-TgANT was broken and the supernatant and inclusion bodies were separated, and protein level was measured by Coomassie brilliant blue method. The results showed that the recombinant protein was mainly expressed in the supernatant (Fig 2A). Expression was induced in *E. coli* BL21 by the addition of 1 mM IPTG for 24 h at 37°C, and the resulting protein was purified by using the His-Binding-Resin and imidazole (Fig 2B). ICR mice were immunized with 100 μg each of recombinant protein and Freund complete adjuvant, followed by four boosts of recombinant protein in incomplete adjuvant. Followed, Mouse anti-TgANT antiserum was used at 1:200, 1: 500, 1:1000 dilution to carry out the western blot and IFA with parasite lysates. However, neither WB (data not saved) nor IFA (Fig 2C) could detect endogenous TgANT signal. Therefore, the polyclonal antibody we prepared was failed and could not be used to detect TgANT protein.

**Fig2. Preparation of TgANT polyclonal antibody**

(A) The IPTG-induced *E. coli* harboring peSUMO-TgANT was broken and the supernatant and inclusion bodies were separated, and protein level was measured by Coomassie brilliant blue method. (B) His-Binding-Resin and imidazole were used to purify the TgANT recombinant protein, and eluting with 50%-80% concentration of imidazole can obtain pure recombinant protein. (C) IFA was used to detect the TgANT recombinant protein. Scale bars: 2 μm

3. For Figure 3A, in the presence of unlabeled ATP and ADP, the 32P-ATP-preloaded *E. coli* transports out both ATP and ADP, but the ratio seems the same regardless of the addition of ATP or ADP extracellularly. In the presence of ATP, ADP will be transported out more than ATP and vice versa. Please explain why no difference is observed between Lane 2 and 3.

**Response：**We also observe that no clear difference is observed between Lane 2 and 3. In order to further verify the reliability of the results, we repeated the experiment (Fig3), but the two results were basically the same. In the new experimental results, the addition of ADP (+ADP) seems to cause more ATP efflux. The latest research showed that the ER ATP/ADP transporter (AXER) in mammals does not behaves as a strict ATP/ADP antiporter, like the ATP/ADP transporter in mitochondria[2]. PfUGT, the homologous protein of TgANT in Plasmodium, not only has affinity for ATP and ATP, but also has a certain affinity for CTP (This part has been added in the manuscript). We speculate that no clear difference between Lane 2 and 3 may be due to the different affinities for ATP/ADP between the inner and outer sides of the TgANT membrane.

**Fig3 Back exchange experiments with *E. coli* cells.**

*E. coli* cells expressing TgANT were preloaded with 50nM radioactive [α-^32^P] ATP, followed by carried out back exchange experiment with ATP, ADP. The supernatant of the broken *E. coli* cells was used to detect the metabolic activity

4. Fig 3B. Controls without the addition of ATP and ADP are needed as a reference.

**Response：** We carried out the back exchange experiments using [α-^32^P] ATP again (Fig 4), and unlabeled ATP and ADP as a reference. Results from three independent assays.

Fig 4 *E. coli* cells harboring the plasmid encoding TgANT were incubated with 50 nM [α-^32^P] ATP for 10 min. At the indicated time points, efflux was induced by the addition of fifty-fold unlabeled ATP **(A)** or ADP **(B)**. Means ± s.e.m., three independent assays.

5. Line 234-236, why does the N-terminally HA-tagged TgANT fail to be detected by WB? The tag facing the ER lumen is degraded? If so, why did IFA work?
**Response：**This is also a problem we have always wanted to solve. TgANT is an ER membrane protein with ten transmembrane domains. Compared with soluble proteins in the cytoplasm, membrane proteins are more difficult to extract. We used TMHMM to predict the structure of TgANT and no signal peptide was found at N-terminus, while the KKQC at C-terminus may be an ER localization signal. Therefore, the Ty epitope we inserted into the N-terminal of the iTgANT strain was not degraded. IFA detection of Ty or HA epitopes requires permeabilization with Triton X-100. Triton X-100 can improve the permeability of eukaryotic cell membranes and enable better binding of antibodies to antigens. Therefore, Ty or HA epitopes can be detected by IFA.

6. Fig 4C. It should be cautious about stating that the expression of TgANT is completely abolished at 24-h post-ATc treatment. There are still some stainings of HA by IFA.
**Response：**We have revised this description in our manuscript.

The expression of the TgANT after ATc treatment was monitored by immunofluorescence (IFA) using the specific antibody of Ty epitope, which revealed that the TgANT was *almost* undetectable after 24h treatment with 0.5 μg/ml ATc.

**In addition, the initial concentration of ATc used in our experiments was 2mg/mL (TaKaRa, 631310), and it was diluted 1:4000, so the final concentration of ATc was 0.5μg /mL. We have revised in our manuscript.**

7. Fig 6-8 showed strong evidence that a conserved peptide motif is key for TgANT function. The author claimed that the TgANT transports ATP into the ER lumen for protein biosynthesis. However, there is a lack of in vivo evidence to support this critical information from this manuscript. I suggest that the authors consider introducing an ATP/ADP biosensor to quantitatively compare the ER's energy status between WT and KO, such as ATP level and ATP/ADP ratio, or use other biochemical strategies. You can refer to the following paper for more information about biosensors. <https://www.ncbi.nlm.nih.gov/pmc/articles/PMC3852917/>

**Response：**This is a very constructive proposal; It would be a good complement to our results if the ATP status in the ER could be detected. We have tried to use the ATP sensor (ERAT4.01)[3] located in the ER to detect the content of ATP in the ER after the TgANT depletion. Calreticulin signal sequence (MLLPVPLLLGLGAAAD) was fused to the N-terminus, a classic ER-retention signal KDEL sequence was added to the C-terminus of the ATP sensor (ERAT4.01), which can guide the sensor to the ER for expression. At present, this method has been applied in a variety of cells to detect changes in ATP in the endoplasmic reticulum, and it is a specialized ATP sensor located in the ER[3, 4]. To detect the ER's energy status in the ER after TgANT depletion, we inserted ERAT4.01 into the UPRT site of the iTgANT strain. However, after chloramphenicol (CAT) screening, ERAT4.01 was expressed in the cytoplasm but not in the ER (Fig 5A). We next constructed a new ER ATP sensor by replacing the HDEL at the C-terminus of ERAT4.01 with KKQC (C-terminus sequence of the TgANT gene), and replacing the calreticulin signal sequence at the N-terminus of ERAT4.01 with the 63 amino acids at the N-terminus of the TgANT (The 63 amino acids at the N-terminal of the TgANT gene can guide the AXER to the ER of *T. gondii*). Unfortunately, the new ER ATP sensor is still expressed in the cytoplasm, not the endoplasmic reticulum (Fig 5B). This part of the results we have described in the Discussion section of our submitted manuscript.

**Fig5. Localization detects of ER** **ATP sensor in the *T. gondii***

(A) The iTgANT strains expressing ER ATP sensor were grown on cell slide for 24 hours, the nuclei were stained with Hoechst, and the localization of ER ATP sensor was determined under excitation light of 488 nm. (B) The iTgANT strain containing the modified ER ATP sensor was directly placed on the glass slide, and the localization of the sensor in *T. gondii* was observed using 488 nm excitation light. Scale bars: 2 μm

8. For Fig 9, in panel A-C, the translation is arrested in the TgANT knockout via upregulated phosphorylation of TgIF2a. Since the replication is reduced dramatically in the KO, it is not surprising to see these phenotypes. There are a few possibilities that ER stress can cause, such as protein folding defects, abnormal ER-associated protein degradation, and ER stress-induced apoptosis. The author quantified higher apoptosis in the KO than WT. Due to the upregulation of eIF2a, the Ca2+ concentration in the ER should be decreased, and the ER-mitochondria contact sites get increased. Further, the Ca2+ is transferred to mitochondria, which induces apoptosis. The authors need to test this hypothesis in the TgANK KO to understand the molecular mechanism by which the deletion of TgANT triggers apoptosis.

**Response：**

Apoptosis is divided into endogenous apoptotic pathway (mitochondrial apoptotic pathway), extrinsic apoptotic pathway (death receptor pathway), and apoptotic pathways induced by ER stress.

At the mitochondria, mitochondrial outer membrane permeabilization (MOMP) is a pivotal event during apoptosis[5]. Following MOMP, pro-apoptotic factors (apoptogens) normally found in the space between the inner and outer mitochondrial membranes are irreversibly released into the cytoplasm[6]. Activation of the intrinsic or mitochondria-dependent pathway leads to cytochrome c release, apoptosome formation, and caspase activation[7]. In the extrinsic pathway, death ligands: FasL (CD95 L), tumor necrosis factor-α (TNF-α), TNF-related apoptosis-inducing ligand (TRAIL) or TNF ligand superfamily member 10 (TNFSF10) interact with their respective death receptors: Fas (CD95), TNF-receptor 1 (TNFR1), death receptor 4 (DR4,) and DR5 (TRAILR2)[8]. Extracellular ligand binding to death receptors triggers the extrinsic pathway that directly activates caspases or translocates tBid to the mitochondria, which in turn leads to activation of the intrinsic pathway[9, 10].

Both apoptotic signaling pathways converge at the level of effector caspases, such as caspase-3 and caspase-7[11, 12]. The transduction of apoptotic signaling can lead to the opening of the permeability transition pore (PTP) in the mitochondrial inner and outer membranes. The PTP has a role in regulating mitochondrial membrane potential and the release of cytochrome c. In the cytosol, released cytochrome c, Apaf1, and procaspase-9 from the mitochondria interact to form the apoptosome that drives the activation of caspase-3[13]. Caspase-3 cleaves many substrate proteins, some of which are endonucleases including caspase-activated DNase (CAD) that translocates to the nucleus to cleave DNA into internucleosomal fragments [14].

In mammals, pathological conditions or microbial infection caused by ER stress is overwhelming, these processes are mainly regulated by three factors: kinase RNA-like ER kinase (PERK), activating transcription factor 6 (ATF6), and inositol-requiring enzyme 1 (IRE1). They restore endoplasmic reticulum homeostasis by inhibiting mRNA transcription, enhancing ER folding capacity, and ERAD (ER-assisted degradation). However, if the stress state of the ER cannot be relieved, continuous ER stress can cause cell apoptosis[15]. Ire1 phosphorylates JNK, leading to inhibition of Bcl-2 activity and activation of Bim, and recruits, releases, and activates procaspases in the cytosol. Induction of CHOP via XBP1, ATF6, or ATF4, down-regulates prosurvival Bcl-2-family members, the balance between proapoptotic and antiapoptotic Bcl-2-family members is disturbed, with activation of the intrinsic apoptotic pathway. This is the third non-classical apoptotic pathway in mammals.

Previous studies have shown that deletion of the ER ATP/ADP transporter in mammals results in the diffusion of calcium ions from the ER into the cytoplasm via Sec61. Elevated cytosolic calcium concentration regulates cytosolic ATP production through calcium/calmodulin dependent kinase kinase 2 (CAMKK2), AMPK, and PF2k[4, 16]. However, calcium ions in the cytoplasm can not only promote glycolysis to generate more ATP but also activate the mitochondrial apoptosis pathway[17, 18]. Massive and/or a prolonged accumulation of Ca^2+^ in the mitochondria can lead to the opening of the permeability transition pore (PTP) in the mitochondrial inner and outer membranes. Followed, released cytochrome c, Apaf1, and procaspase-9 from the mitochondria interact to form the apoptosome that drives the activation of caspase-3, leading to apoptosis.

However, in apicomplexan, the exploration of the apoptosis pathway is a challenging task. Although the signature feature of apoptosis was observed and identified in the protozoa, including shrinkage and rounding of the body shape, DNA fragmentation, and nucleus condensation. But most of the genes that have been identified as participating in the apoptosis pathway of metazoans are missing in the genomes of parasitic protozoans, such as ATF6, CHOP, and XBP, especially the caspase family proteins that play an important role in the process of apoptosis [19-21]. Hence, the apoptosis pathway of protozoa may differ from that of mammals. More comprehensive and systematic work may be required to uncover the mechanism of apoptosis in *T. gondii.*

Since a variety of apoptosis pathways are involved in the reduction of mitochondrial membrane potential, we examined the mitochondrial membrane potential of iTgANT strains after ATc treatment. The results showed that although the membrane potential decreased after ATC treatment, but there is no statistically significant difference compared with the untreated group (Fig 6). Although previous studies have shown that the depletion of the ER ATP/ADP transporter leads to the efflux of Ca^2+^ from the ER[4, 16]. But Ca^2+^ in *T. gondii* have multiple functions, not just affecting mitochondria. Therefore, it may not be comprehensive to explore the apoptosis pathway of *T. gondii* only from the changes of mitochondria caused by Ca^2+^ efflux. Furthermore, we believe that there may be many reasons for the growth defects in *T. gondii* caused by TgANT deletion, and the fragmentation of nuclear DNA we observed is just one of them. Next, we may investigate why TgANT depletion causes severe growth defects in *T. gondii*, but apoptosis of iTgANT strain is not the focus of our study for now.

**Fig6. Mitochondrial membrane potential detection with JC-1**

When the mitochondrial membrane potential is high, JC-1 aggregates in the mitochondrial matrix to form J-aggregates, which can produce red fluorescence; When the mitochondrial membrane potential is low, JC-1 cannot aggregate in the mitochondrial matrix, and JC-1 is a monomer at this time, which can produce green fluorescence. The relative ratio of red and green (590/480) fluorescence is often used to measure the mitochondrial membrane potential.

Reviewer #3: (No Response)

**Part III – Minor Issues: Editorial and Data Presentation Modifications**
Please use this section for editorial suggestions as well as relatively minor modifications of existing data that would enhance clarity.

Reviewer #1: ER localisation.
Lines 140-150: The authors describe two different tagging strategies that were used to localise TgANT, in two different strains. It is not clear why the authors do not show the data for both strategies.

**Response：**Two different tagging strategies demonstrate that TgANT is localized in the ER. To avoid repetition, we only show one result, the results for adding a 10×HA tag to the C-terminus are as follows (Fig7).

**Fig7. Localization detects of ER ATP sensor in the *T. gondii***

TgANT-10×HA parasites were subjected to immunostaining using antibodies against the HA (red) tag along with SERCA (ER), The result shows that TgANT was complete co-localization with the ER-resident protein SERCA. Scale bars: 5 μm.

Fig. 1C: A plasma membrane marker and a DIC image would be helpful to demonstrate the ER localisation of TgANT.

Response：We reworked the localization experiments using the inner membrane complex (IMC) and plasma membrane marker (GAP45) antibody and the results are as follows (Fig8).

**Fig8. Localization detects of ER ATP sensor in the *T. gondii***

TgANT-HA parasites were subjected to IFA using antibodies against the HA (red) tag along with IMC, GAP45 and SERCA (ER). The result shows that TgANT was complete co-localization with the ER-resident protein SERCA, but not co-localization with plasma membrane of *T. gondii*. Scale bars: 5 μm.

The antibody used to label SERCA is not named and its source is not provided. It is not stated whether this antibody is specific to the SERCA from T. gondii or not. These issues should be fixed.

**Response：**The SERCA antibody is a polyclonal antibody prepared in our laboratory. The nucleotides 1117 to 2415, corresponding to amino acid residues 373 to 805 of the [TGGT1_230420](https://toxodb.org/toxo/app/record/gene/TGGT1_230420) (sarco/endoplasmic reticulum Ca^2+^-ATPase) were used to make polyclonal antibody and the specificity has been proven[22]. In addition, we have added information on all antibodies used in our study in the Materials and methods of the manuscript

**Transport studies.**

Information should be provided on whether there were an equal number of viable bacteria in the IPTG-induced and non-induced conditions in the ATP transport experiments.
**Response：**All the experiments of take up radioactively labeled nucleotide were used equal amounts of *E. coli*, we double checked the Materials methods section and indeed the description of *E. coli* uninduced by IPTG was missing, we have supplemented in the Materials methods section.

It would be useful to test an additional radiolabeled substrate as a control – one that is not a substrate of TgANT but that is taken up by bacteria (to check that IPTG induction/TgANT expression does not non-specifically increase the uptake of unrelated compounds).

**Response：**This is a very constructive comment. We initially tried to verify that TgANT expression did not affect *E. coli* uptake of substrates such as glucose. However, due to the limitations of experimental materials and experimental detection conditions, we were unable to complete this research. But this question has been validated in an initial study using *E. coli* to verify the transport capacity of the *Arabidopsis* plastidic ATP/ADP transporter. Their results demonstrate that IPTG induction did not promote import of other phosphorylated or unphosphorylated ([^14^C] Glc6P and [^14^C] Glc) metabolites into the bacterial cell indicating the specificity of [α-^32^P] ATP transport[23].

The Michaelis-Menten curves from which the Km and Vmax values were derived should be shown. As it currently stands, a *Km* and *Vmax* are cited in the text, with no error and no detail anywhere in the manuscript about the conduct of the experiments from which these values were derived.

**Response：**We are very sorry, we need to explain that this is a wrong data, our existing experiments cannot get the *Km* and *Vmax* of TgANT. Previous results have shown that the substrate saturation of the ER ATP/ADP transporter (AXER) exceeds 100 μM in mammals and that of ER-ANT1 in plants exceeds 300 μM. Due to the limited amount of [α-^32^P] ATP, we did not have enough [α-^32^P] ATP for the corresponding experiments. We attempted to use a proportional mixture of [α-^32^P] ATP and Non-radioactive ATP for detection, and then convert the obtained results to calculate the absorption curve of TgANT. However, as the content of non-radioactive ATP increased, the amount of radioactive ATP absorbed by *E. coli* gradually decreased, and the value obtained by the liquid scintillation counter gradually decreased, the radioactivity is almost undetectable, resulting in huge errors in the data. Our results show that substrate saturation of TgANT still exhibits a linear curve in the 1μM [α-^32^P] ATP range. Therefore, we need to delete the relevant data in the manuscript.

Does ‘three-time repetition’ (used numerous times throughout the manuscript) refer to technical replicates or independent experiments performed on different days? This should be clarified. Independent experiments are required for the transport studies.
**Response：**We are very sorry for the confusing question about existence in statistics. Invasion efficiencies experiment and all intracellular replication assays in the manuscript were performed in three or more independent experiments, and one of the independent replicates are presented in the manuscript. The data statistics of the plaque experiment come from three independent replicates, and the number of plaques selected from each independent replicate experiment is not less than thirty. All the [α-^32^P] ATP transport studies, and apoptosis experiment were performed not less than three independent experiments. we have revised in each legend of the manuscript.

PfUGT.

The authors state that PfUGT also functions as an ATP/ADP transporter but do not show the data. In the Discussion, they state: “our data proved that PfUGT (PfANT) in P. falciparum is also an ATP/ADP transporter”. Perhaps the authors plan to submit a separate study on PfUGT, in which case this would be good to clarify. Otherwise, it is not clear why the authors have chosen not to present these very important data that would be of great interest to the malaria field. With a functional assay for PfUGT, it would also be interesting to test whether ganaplacide affects the function of the protein.
**Response：**We have added relevant experimental data for PfUGT to the manuscript.

Clarity and additional details.

Overall, the manuscript needs editing to improve clarity.

Line 182: should Fig S2A be Fig S1A?
**Response：**It should be S1A, we revised in the manuscript

Fig 3A: it would be helpful to state what is in each of the lanes on the Figure itself or in the Figure legend.
**Response：**We have added information in each of the lanes.

Fig 4C: what is ALD? This should be explained in the legend, and the source of all the antibodies used in the study, including the anti-ALD antibody, should be provided.
**Response：**We have added information on all antibodies used in our study in the Materials and methods of the manuscript.

Was iTgANT1 made in a Delta-ku80 background (i.e. is the strain the authors call ‘TATi’ TATi-Delta-ku80? The sources/references for the parent strains used in the study should be provided.
**Response：**We have added information of transgenic parasites used in this study in “S4 Table”. iTgANT was made in TATi strain but not TATi-Delta-ku80[24].

The titles of Figures 6 and 7 need changing as the E. coli uptake experiments are shown in Figure 6 not Figure 7.

**Response：**We have made corrections in our manuscript.

Line 587 – should this be F37V (the resistance-conferring mutation in PfUGT)? The relevant studies about its role in resistance should be cited. There are no references at all in the relevant paragraph of the Discussion, which should also include references to the statements pertaining to Arabidopsis.

**Response：**We are very sorry for the erroneous description in our manuscript, the mutation site in PfUGT(we propose to name PfANT) is F37V and the corresponding site in TgANT is F81V. we have revised in the manuscript

There is no literature supporting the PfUGT (we propose to name PfANT)[25] and Arabidopsis[26] in the manuscript, this is an oversight and we have inserted the reference in the Discussion section.

**Lines 591 – 592: editing is required, the meaning is unclear.
Response：**We have revised in the manuscript. In *P. falciparum*, although the mutate of PfUGT (F37V) lead to resistance to GNF179 and KAF156, but this mutation also makes it difficult to survive in animals. Parasites that harbor mutate of PfUGT proliferate at a reduced rate. The researchers speculate that these low fitness mutants might not survive the competition for nutrients in a polyclonal environment such as the human body, or may be more readily cleared by the immune system.

It is not clear exactly how the ER-localising AXER fusion protein was made – more detail, including exactly which residues of both AXER and TgANT were present in the fusion protein, is needed.
**Response：**The description in this part is indeed lacking in relevant information, and we have further supplemented the relevant details in “Table S5”

Fig 9B: it is not clear what was loaded into which lanes (e.g. what is the difference between lanes 2 vs 4 and 3 vs 5 – proteins from intracellular vs extracellular parasites?

**Response：**We are very sorry that we left out key information of the Fig 9B. We have added the corresponding annotations, the first two lanes are representing the extracellular tachyzoites and the last two lanes are representing the intracellular tachyzoites

Reviewer #2: 1. The authors gave a comprehensive literature review for ER ANT and AXER but barely mentioned the ER stress studies in Toxoplasma and other pathogens. It would be great to see this information in the Introduction.

**Responses:** The literature review of ER stress in *T. gondii* is mainly placed in the last part of the manuscript, which is not the focus of our study. Therefore, this part of the literature review is not included in the introduction part of the manuscript.

2. Line 141, a typo, C-C-terminus
**Responses:** This is a wrong description, the correct is "C-terminus", which we have revised in the manuscript

3. Line 296, a typo, potential

**Responses:** we have revised in the manuscript.

Reviewer #3: Abstract, line 16: ‘lumens of the endoplasmic reticulum (ER) is the subcellular sites’ should be lumen and should be site (not the plurals). There are many such minor grammatical issues, which of course I will not all highlight.

**Responses:** We are very sorry for the grammatical issues in our manuscript, we have made corrections in the new manuscript.

In the abstract, line 22, please introduce the acronym TgANT1 after ‘an ATP/ADP transporter’.

**Responses:** ATP/ADP transporter belong to Adenine Nucleotide Transporter, so we named it TgANT according to the naming method in plants, and can be distinguished from mitochondrial ATP/ADP transporters (AACs). We have summarized in the manuscript.

Line 41: ‘ADP/ADP transporter (TgANT)’ should be ATP/ADP transporter. Also, throughout the manuscript, space before opening brackets is missing. Could be an issue with the software.

**Responses:** This is our mistake; we have revised in the manuscript.

Line 58: ‘required/needed’ either word should be fine.

**Responses:** We have revised in the manuscript.

Line 92-93: ‘it only caused the activity of the BIP protein to decrease and slight cell growth slowed down.’ The BIP protein is not introduced and I do not understand this statement.

**Responses:** The description in this part is not clear enough and we have made changes in the manuscript

Line 98-99: Caenorhabditis elegans in italics as species name please. (also line 349)

**Responses:** We have revised in the manuscript.

Line 115-116: ‘we heterologous expression TgANT in E. coli, this system has previously…’.Multiple grammatical errors in this sentence.

**Responses:** We have revised in the manuscript. For functional characterization of TgANT, we expression TgANT in *E. coli*, this system has been shown to functionally integrate several membrane proteins into the bacterial cytoplasmic membrane

Line 124: ‘Blastp analysis revealed only three sequences encoding putative solute carriers family protein’. I do not think this can be correct as stated. However, it is quite possible that BLASTP analysis identified only three genes (within the set parameters), which were putative solute carriers.

**Responses:**

We strongly agree with the view that homology analysis needs to set a certain Expectation value in *ToxoDB*, and our initial search for proteins homologous to ER-ANT1 and AXER may not be comprehensive. The genome of several organisms including yeast, plants and mammals contains multiple genes encoding the mitochondria ADP/ATP transporter, and have high homology [27-30]. To determine whether there are multiple genes encoding the ER ADP/ATP transporter of *T. gondii*, we used protein sequence of TgANT to find out whether there is a homologous protein in *T. gondii* after we verified the function of TgANT. But we did not find another homologous protein. Our initial approach to finding ER ATP/ADP transporters had some loopholes, but we believe that there is only a single ER ATP/ADP transporter in *T. gondii*, which we will add to the ‘Discussion’ in our manuscript.

Line 130. ‘experiments show that they are located in the mitochondria instead of the ER.’ This needs clarification and perhaps some references. Which experiments, described where?

**Response：**Since ER-ANT1 has high homology (more than 60%) with the mitochondrial ATP/ADP transporter of Arabidopsis[1], these three proteins in *T. gondii* found by Blastp analysis are also located in mitochondria, rather than located in the ER. At present, the functions of these three proteins are still under study in our laboratory, but the localization results have confirmed that these three proteins are located in mitochondria, not in the ER (Fig. 1). We have summarized in the manuscript which gene is named TgANT.

Line 136: ‘C-terminally dilysine motif shown in other proteins to limit exit from the ER (Fig 1B).’ I fail to see this motive in Figure 1B. It is possible Figure 1A is meant instead, but the reproduction of the small font size is such (in my copy) that I can’t make it out.

**Responses:** This is a very good suggestion, the C-terminal polylysine motif is indeed only seen in (Fig 1A). we have revised in the manuscript. We will improve the quality of the Figure to better see all amino acid sequences

Line 139-140 ‘…but it was not available’. Sorry, this is not clear. You tried to prepare an antibody but it was not available? Do you mean ‘not successful’? Or the epitope was not available for binding of antibodies? Please clarify.

**Responses:** The description in our manuscript is confusing and we have revised it in the new manuscript. We tried to prepare TgANT polyclonal antibodies using the three peptide fragments outside the transmembrane region of TgANT exposed to the cytoplasm. However, the polyclonal antibody we prepared does not work and could not detect TgANT protein, either by Western blot or IFA.

Line 141: I am not familiar with the description C-C terminus.

**Responses:** This is a wrong description, the correct is "C-terminus", which we have revised in the manuscript

Line 150: co-localized, not ‘co-localization’.

**Responses:** we have revised in the manuscript

Figure 1 legend: ‘The nitrogen termini and carboxy termini face the ER lumen, the double lysine motif(-KKQC) near the C-terminally of TgANT’ should be ‘The nitrogen terminus and carboxy terminus face the ER lumen, the double lysine motif (-KKQC) is located near the C-terminal end of TgANT.’

**Responses:** we have revised in the manuscript

Line 168-170: ‘Western blot and IFA. As expected, the TgANT was expressed and inserted into the bacterial membrane(Fig 2A).’ Is this Western blot shown anywhere?

**Responses:** The expression of TgANT on the outer membrane of *E. coli* is the premise for its ability to transport ATP or ADP. As a prokaryotic organism, *E. coli* does not have many organelles including the ER, while TgANT has ten transmembrane domains and difficult to express in large quantities in *E. coli*. In other research[1, 16, 31], although the outer membrane of *E. coli* was isolated for Western Blot experiments to prove that foreign proteins can be expressed on the outer membrane of *E. coli*, we believe that this method is still difficult to avoid the influence of proteins in the cytoplasm of *E. coli.* Therefore, we first tried to detect whether TgANT could be expressed on the E. coli membrane by IFA, which is the most direct method to verify whether TgANT is expressed on the membrane. Our results clearly show that TgANT can be expressed on the membrane after IPTG induction, which is a prerequisite for its ability to absorb radioactive ATP. We also tried to isolate the outer membrane of *E. coli* induced by IPTG, but the results were not satisfactory, and no obvious TgANT signal could be observed by Western Blot. We thought that this might be related to the low expression of TgANT in *E. coli*.

In our manuscript, we would like to emphasize that we demonstrated TgANT is expressed on *E. coli* membranes by IFA. I think mention of Western Blot is really inappropriate, we didn't show experimental results for Western Blot, which would be confusing. We therefore propose to revise the statement in the manuscript as follows: After isopropyl-β-D-thiogalactopyramoside (IPTG) induction, we investigated whether the TgANT is integrated into the plasma membrane using IFA. As expected, the TgANT was expressed and inserted into the bacterial membrane (Fig 2A).

Line 172-175: ‘The import of radioactively labelled [α-32P]ATP by the recombinant TgANT into E. coli cells displayed typical Michaelis-Menten kinetics, with apparent Km values of 3.719 μM and Vmax of 6.974 nmol/min·mg for ATP.’ The determination of the Km is important but the data are not shown anywhere. Is this a single determination? If the average of multiple experiment, SD or SEM should be given.

**Responses:** ‘The import of radioactively labelled [α-^32^P]ATP by the recombinant TgANT into E. coli cells displayed typical Michaelis-Menten kinetics, with apparent Km values of 3.719 μM and Vmax of 6.974 nmol/min·mg for ATP.’ We are very sorry, we need to explain that this is a wrong data, our existing experiments cannot get the *Km* and *Vmax* of TgANT. Previous results have shown that the substrate saturation of the ER ATP/ADP transporter (AXER) exceeds 100 μM in mammals and that of ER-ANT1 in plants exceeds 300 μM. Due to the limited amount of [α-^32^P] ATP, we did not have enough [α-^32^P] ATP for the corresponding experiments. We attempted to use a proportional mixture of [α-^32^P] ATP and Non-radioactive ATP for detection, and then convert the obtained results to calculate the absorption curve of TgANT. However, as the content of non-radioactive ATP increased, the amount of radioactive ATP absorbed by *E. coli* gradually decreased, and the value obtained by the liquid scintillation counter gradually decreased, the radioactivity is almost undetectable, resulting in huge errors in the data. Our results show that substrate saturation of TgANT still exhibits a linear curve in the 1μM [α-^32^P] ATP range. Therefore, we need to delete the relevant data in the manuscript.

Line 175-176: ‘the uninduced E. coli was not able to absorb ATP or ADP at significant rates (Fig 2B).’ Actually the slope of the line in Figure 2B looks non-zero, probably a significant uptake albeit of course very much lower than the cells expressing TgANT1.

**Responses:** We also noticed this fact. we think that although TgANT expression in *E. coli* requires IPTG induction, but in fact, *E. coli* without IPTG can still express a very low content of TgANT. Because TgANT with HA epitope can still emit extremely weak fluorescence through IFA experiment in *E. coli* without IPTG.

Line 179: ‘The ability of E. coli to uptake radioactive ATP..’ take up, not uptake (uptake is not a verb).

**Responses:** we have revised in the manuscript

Line 184-185: ‘PfUGT also does not have the UDP-galactose transport capacity but has a strong affinity for ATP and ADP(Data not shown).’ Sorry but this is an important claim and requires the data to be shown - or the claim not made - as far as I am concerned. Same for lines 585-586 in the Discussion: ‘our data proved that PfUGT (PfANT) in P. falciparum is also an ATP/ADP transporter, not a UDP-galactose transporter’. I do not see that as proven. Further in lines 586-588: ‘the mutation of one amino acid (F81V) of PfANT in P. falciparum resulted in resistance to two antimalarial imidazolopiperazines, KAF156 and GNF179’ Again, I do not see the evidence upon which this conclusion is based.

**Responses:**

We have added relevant experimental data for PfUGT to the manuscript.

There is no literature supporting the association of PfUGT (we propose to name PfANT) mutations in KAF156 and GNF179 resistance in the manuscript, this is an oversight and we have inserted the reference in the Discussion section.

(Lim MY, LaMonte G, Lee MCS, Reimer C, Tan BH, Corey V, Tjahjadi BF, Chua A, Nachon M, Wintjens R, Gedeck P, Malleret B, Renia L, Bonamy GMC, Ho PC, Yeung BKS, Chow ED, Lim L, Fidock DA, Diagana TT, Winzeler EA, Bifani P. UDP-galactose and acetyl-CoA transporters as Plasmodium multidrug resistance genes. Nat Microbiol. 2016 Sep 19;1:16166. doi: 10.1038/nmicrobiol.2016.166. PMID: 27642791; PMCID: PMC5575994.)

Table 1: The column heading ‘Inhibit (%)’ should be’ uptake (% of control)’.

**Responses:** we have revised in the manuscript

Table 1 legend: ‘a mixture of 25 nM [α-32P] ATP and One-hundred-fold potential substrates (Non-radioactive) was used’ would read better as ‘a mixture of 25 nM [α-32P] ATP and non-radioactive potential substrate at one-hundred-fold concentration was used’

**Responses:** we have revised in the manuscript

Figs 3B,C: The outflow of ATP and ADP is partial, rapidly plateauing. Is the rest intracellular 32P-AMP (and thus trapped inside the cells) or is there a different explanation?

**Responses:** Due to metabolic activity of *E. coli* cells，[α-^32^P] ATP was converted into labeled ADP, AMP, and inorganic phosphate (Pi), This has been confirmed in previous studies[1, 16, 31]. Therefore, ten minutes after *E. coli* absorbs [α-^32^P] ATP, the [α-^32^P] ATP in *E. coli* has been partially decomposed into [α-^32^P]ADP, [α-^32^P] AMP and Pi. Additional non-radioactive ATP or ADP can chase radioactive [α-^32^P] ATP or [α-^32^P]ADP in *E. coli*, but the [α-^32^P] AMP cannot be chase from *E. coli.* Therefore, the radioactive signal in *E. coli* gradually becomes stable.

Line 234-236: ‘Because TgANT has multiple transmembrane domains and both the N-terminal and C-terminal face the ER lumen, it was failed to detect Ty epitope by western blot.’ Please explain. It seems to me that the IFS might have issues with that but the Western blot should work.

**Responses:** This is also a problem we have always wanted to solve. TgANT is an ER membrane protein with ten transmembrane domains. Compared with soluble proteins in the cytoplasm, membrane proteins are more difficult to extract. We used TMHMM to predict the structure of TgANT and no signal peptide was found at N-terminus, while the KKQC at C-terminus may be an ER localization signal. Therefore, the Ty epitope we inserted into the N-terminal of the iTgANT strain was not degraded. IFA detection of Ty or HA epitopes requires permeabilization with Tirton X-100. Triton X-100 can improve the permeability of eukaryotic cell membranes and enable better binding of antibodies to antigens. Therefore, we think this is the reason why WB did not work, but IFA was able to detect Ty or HA epitopes.

I see references to Figure S1B and S1C but not S1A.

**Responses:** we have revised in the manuscript.

Line 517: ‘If TgANT is only the UDP-galactose transporter, then theoretically, the lack of TgANT will not cause the fatal growth defect of T.gondii.’ The reasoning is astute and likely correct - but unproven. As such a phrasing like ‘...would likely not cause a fatal growth defect in T. gondii.’ is more appropriate.

**Responses:** We fully agree with this revision, which makes our inferences more accurate. we have revised in the manuscript

Line 539-541: ‘Since these two highly conserved domains exist in *T. gondii*, *Plasmodium*, *HeLa cells*, and *C.elegans*, we speculate that the ER ATP/ADP transport mechanism is shared among different species.’ That is overstating a bit. Even if it can be taken that these domains likely represent a signature for an ATP / ADP exchanger, the localisation is a matter of other sequences. The authors do not make the case that these domains are necessarily of ER-located transporters.

**Responses:** We fully agree with this point that it is indeed inappropriate to make this inference in our manuscript. We have no experimental support that the genes we selected for homologous alignment must be located in the ER, and we need to make revisions. Since these two highly conserved domains exist in T. gondii, Plasmodium, HeLa cells, and C.elegans, we speculate that the ER ATP/ADP transport mechanism is shared among different species.

We propose the following modifications: ‘Since these two highly conserved domains exist in *T. gondii*, *Plasmodium*, *HeLa cells*, and *C.elegans*, We speculate that this transport mechanism may be applicable in multiple species.’

Line 575: ‘Unlike the ER ATP/ADP transporter in mammals, Apicomplexan Parasites and nematodes’ no need to capitalize Apicomplexan and Parasites.
Discussion: In my opinion the discussion about Arabidopsis, especially regarding ROS, can be shortened.

**Responses:** we have revised in the manuscript

1.	Leroch M, Neuhaus HE, Kirchberger S, Zimmermann S, Melzer M, Gerhold J, et al. Identification of a novel adenine nucleotide transporter in the endoplasmic reticulum of Arabidopsis. Plant Cell. 2008;20(2):438-51. doi: 10.1105/tpc.107.057554. PubMed PMID: WOS:000256415400018.

2.	Schwarzbaum PJ, Schachter J, Bredeston LM. The broad range di- and tri-nucleotide exchanger SLC35B1 displays asymmetrical affinities for ATP transport across the ER membrane. J Biol Chem. 2022;298(4):101537. Epub 2022/01/19. doi: 10.1016/j.jbc.2021.101537. PubMed PMID: 35041824.

3.	Vishnu N, Khan MJ, Karsten F, Groschner LN, Waldeck-Weiermair M, Rost R, et al. ATP increases within the lumen of the endoplasmic reticulum upon intracellular Ca2+ release. Mol Biol Cell. 2014;25(3):368-79. doi: 10.1091/mbc.E13-07-0433. PubMed PMID: WOS:000339628100006.

4.	Yong J, Bischof H, Burgstaller S, Siirin M, Murphy A, Malli R, et al. Mitochondria supply ATP to the ER through a mechanism antagonized by cytosolic Ca(2). eLife. 2019;8. Epub 2019/09/10. doi: 10.7554/eLife.49682. PubMed PMID: 31498082; PubMed Central PMCID: PMCPMC6763289.

5.	Goldstein JC, Waterhouse NJ, Juin P, Evan GI, Green DR. The coordinate release of cytochrome c during apoptosis is rapid, complete and kinetically invariant. Nature cell biology. 2000;2(3):156-62. Epub 2000/03/09. doi: 10.1038/35004029. PubMed PMID: 10707086.

6.	Green DR, Kroemer G. The pathophysiology of mitochondrial cell death. Science. 2004;305(5684):626-9. Epub 2004/08/03. doi: 10.1126/science.1099320. PubMed PMID: 15286356.

7.	Movassagh M, Foo RS. Simplified apoptotic cascades. Heart failure reviews. 2008;13(2):111-9. Epub 2007/12/18. doi: 10.1007/s10741-007-9070-x. PubMed PMID: 18080749.

8.	Ashkenazi A, Dixit VM. Death receptors: signaling and modulation. Science. 1998;281(5381):1305-8. Epub 1998/08/28. doi: 10.1126/science.281.5381.1305. PubMed PMID: 9721089.

9.	Luo X, Budihardjo I, Zou H, Slaughter C, Wang X. Bid, a Bcl2 interacting protein, mediates cytochrome c release from mitochondria in response to activation of cell surface death receptors. Cell. 1998;94(4):481-90. Epub 1998/09/04. doi: 10.1016/s0092-8674(00)81589-5. PubMed PMID: 9727491.

10.	Gross A, Yin XM, Wang K, Wei MC, Jockel J, Milliman C, et al. Caspase cleaved BID targets mitochondria and is required for cytochrome c release, while BCL-XL prevents this release but not tumor necrosis factor-R1/Fas death. J Biol Chem. 1999;274(2):1156-63. Epub 1999/01/05. doi: 10.1074/jbc.274.2.1156. PubMed PMID: 9873064.

11.	Kuribayashi K, Mayes PA, El-Deiry WS. What are caspases 3 and 7 doing upstream of the mitochondria? Cancer biology & therapy. 2006;5(7):763-5. Epub 2006/08/22. doi: 10.4161/cbt.5.7.3228. PubMed PMID: 16921264.

12.	Lamkanfi M, Kanneganti TD. Caspase-7: a protease involved in apoptosis and inflammation. The international journal of biochemistry & cell biology. 2010;42(1):21-4. Epub 2009/09/29. doi: 10.1016/j.biocel.2009.09.013. PubMed PMID: 19782763; PubMed Central PMCID: PMCPMC2787741.

13.	Bock FJ, Tait SWG. Mitochondria as multifaceted regulators of cell death. Nat Rev Mol Cell Biol. 2020;21(2):85-100. Epub 2019/10/23. doi: 10.1038/s41580-019-0173-8. PubMed PMID: 31636403.

14.	Nagata S. Apoptosis and Clearance of Apoptotic Cells. Annual review of immunology. 2018;36:489-517. Epub 2018/02/06. doi: 10.1146/annurev-immunol-042617-053010. PubMed PMID: 29400998.

15.	Song S, Tan J, Miao Y, Li M, Zhang Q. Crosstalk of autophagy and apoptosis: Involvement of the dual role of autophagy under ER stress. Journal of cellular physiology. 2017;232(11):2977-84. Epub 2017/01/10. doi: 10.1002/jcp.25785. PubMed PMID: 28067409.

16.	Klein MC, Zimmermann K, Schorr S, Landini M, Klemens PAW, Altensell J, et al. AXER is an ATP/ADP exchanger in the membrane of the endoplasmic reticulum. Nat Commun. 2018;9. doi: ARTN 3489

10.1038/s41467-018-06003-9. PubMed PMID: WOS:000442909300013.

17.	Marchi S, Patergnani S, Missiroli S, Morciano G, Rimessi A, Wieckowski MR, et al. Mitochondrial and endoplasmic reticulum calcium homeostasis and cell death. Cell Calcium. 2018;69:62-72. doi: <https://doi.org/10.1016/j.ceca.2017.05.003>.

18.	Bravo R, Vicencio JM, Parra V, Troncoso R, Munoz JP, Bui M, et al. Increased ER-mitochondrial coupling promotes mitochondrial respiration and bioenergetics during early phases of ER stress. Journal of cell science. 2011;124(Pt 13):2143-52. Epub 2011/06/02. doi: 10.1242/jcs.080762. PubMed PMID: 21628424; PubMed Central PMCID: PMCPMC3113668.

19.	Iyer LM, Anantharaman V, Wolf MY, Aravind L. Comparative genomics of transcription factors and chromatin proteins in parasitic protists and other eukaryotes. Int J Parasitol. 2008;38(1):1-31. Epub 2007/10/24. doi: 10.1016/j.ijpara.2007.07.018. PubMed PMID: 17949725.

20.	De Silva EK, Gehrke AR, Olszewski K, León I, Chahal JS, Bulyk ML, et al. Specific DNA-binding by apicomplexan AP2 transcription factors. Proc Natl Acad Sci U S A. 2008;105(24):8393-8. Epub 2008/06/11. doi: 10.1073/pnas.0801993105. PubMed PMID: 18541913; PubMed Central PMCID: PMCPMC2423414.

21.	Dixon SE, Stilger KL, Elias EV, Naguleswaran A, Sullivan WJ, Jr. A decade of epigenetic research in Toxoplasma gondii. Mol Biochem Parasitol. 2010;173(1):1-9. Epub 2010/05/18. doi: 10.1016/j.molbiopara.2010.05.001. PubMed PMID: 20470832; PubMed Central PMCID: PMCPMC2886187.

22.	Nagamune K, Beatty WL, Sibley LD. Artemisinin induces calcium-dependent protein secretion in the protozoan parasite Toxoplasma gondii. Eukaryotic cell. 2007;6(11):2147-56. Epub 2007/08/31. doi: 10.1128/EC.00262-07. PubMed PMID: 17766463.

23.	Tjaden J, Schwöppe C, Möhlmann T, Quick PW, Neuhaus HE. Expression of a plastidic ATP/ADP transporter gene in Escherichia coli leads to a functional adenine nucleotide transport system in the bacterial cytoplasmic membrane. J Biol Chem. 1998;273(16):9630-6. Epub 1998/05/23. doi: 10.1074/jbc.273.16.9630. PubMed PMID: 9545295.

24.	Meissner M, Schluter D, Soldati D. Role of Toxoplasma gondii myosin A in powering parasite gliding and host cell invasion. Science. 2002;298(5594):837-40. Epub 2002/10/26. doi: 10.1126/science.1074553. PubMed PMID: 12399593.

25.	Lim MY, LaMonte G, Lee MCS, Reimer C, Tan BH, Corey V, et al. UDP-galactose and acetyl-CoA transporters as Plasmodium multidrug resistance genes. Nature microbiology. 2016;1:16166. Epub 2016/09/20. doi: 10.1038/nmicrobiol.2016.166. PubMed PMID: 27642791; PubMed Central PMCID: PMCPMC5575994.

26.	Hoffmann C, Plocharski B, Haferkamp I, Leroch M, Ewald R, Bauwe H, et al. From endoplasmic reticulum to mitochondria: absence of the Arabidopsis ATP antiporter endoplasmic Reticulum Adenylate Transporter1 perturbs photorespiration. Plant Cell. 2013;25(7):2647-60. Epub 2013/07/19. doi: 10.1105/tpc.113.113605. PubMed PMID: 23860249; PubMed Central PMCID: PMCPMC3753389.

27.	Picault N, Hodges M, Palmieri L, Palmieri F. The growing family of mitochondrial carriers in Arabidopsis. Trends in plant science. 2004;9(3):138-46. Epub 2004/03/09. doi: 10.1016/j.tplants.2004.01.007. PubMed PMID: 15003237.

28.	Palmieri F. The mitochondrial transporter family (SLC25): physiological and pathological implications. Pflugers Archiv : European journal of physiology. 2004;447(5):689-709. Epub 2003/11/05. doi: 10.1007/s00424-003-1099-7. PubMed PMID: 14598172.

29.	Dolce V, Scarcia P, Iacopetta D, Palmieri F. A fourth ADP/ATP carrier isoform in man: identification, bacterial expression, functional characterization and tissue distribution. Febs Lett. 2005;579(3):633-7. Epub 2005/01/27. doi: 10.1016/j.febslet.2004.12.034. PubMed PMID: 15670820.

30.	Smith CP, Thorsness PE. The molecular basis for relative physiological functionality of the ADP/ATP carrier isoforms in Saccharomyces cerevisiae. Genetics. 2008;179(3):1285-99. Epub 2008/06/18. doi: 10.1534/genetics.108.087700. PubMed PMID: 18562646.

31.	Haferkamp I, Hackstein JHP, Voncken FGJ, Schmit G, Tjaden J. Functional integration of mitochondrial and hydrogenosomal ADP/ATP carriers in the Escherichia coli membrane reveals different biochemical characteristics for plants, mammals and anaerobic chytrids. Eur J Biochem. 2002;269(13):3172-81. doi: 10.1046/j.1432-1033.2002.02991.x. PubMed PMID: WOS:000176523900010.
