# Supplementary material for: A new adenine nucleotide transporter located in the ER is essential for maintaining the growth of Toxoplasma gondii
Source: PLoS Pathog. 2022 Jul 5;18(7):e1010665. doi: 10.1371/journal.ppat.1010665 (PMC9286291; doi:10.1371/journal.ppat.1010665)
Supplement: Attachment — Submitted filename: Response to Reviewers.docx [file ppat.1010665.s009.docx]

The reviewers were satisfied with many of the changes to the manuscript, however, two additional issues remain:

1. The corrections listed by R1 should be addressed.

**Response：**We have revised related problems of Reviewer #1 in part- Part III.

2. Statements firmly concluding that ER ATP levels are reduced in mutant/knockdown parasites should be modified because ER ATP levels were not directly measured. The statement at the end of the abstract is fine: "Our results indicate that TgANT is the only ATP/ADP transporter in the ER of *T. gondii*, and the lack of ATP in the ER is the cause of the death of *T. gondii*." The statement in the concluding paragraph should be modified: "The depletion of TgANT leads to the supply failure of ATP in the ER, which ..." Similarly, the section title "The interruption supply of the ER ATP caused the death of *T. gondii*" should be changed and any other similar statements in the manuscript.

**Response：**This is a very constructive comment and we have revised it in our new manuscript.

**Before:** The interruption supply of the ER ATP caused the death of *T. gondii*

**Revised:** The lack of ATP in the ER maybe caused the death of *T. gondii*

**Before:** To further determine the severe phenotypic defect of the iTgANT strain is caused by the interruption of ATP supply in the ER, heterologous supplementation was performed with AXER.

**Revised:** To further determine the severe phenotypic defect of the iTgANT strain is caused by the lack of ATP in the ER, heterologous supplementation was performed with AXER.

**Before:** Additionally, we believe that the depletion of TgANT resulted in the severe growth defects of *T.gondii*, due to the insufficient supply of ATP in the ER.

**Revised:** Additionally, we believe that the depletion of TgANT resulted in the severe growth defects of *T.gondii*, due to the the lack of ATP in the ER.

**Before:** The depletion of TgANT leads to the supply failure of ATP in the ER, which may explain why the iTgANT strain has serious growth defects.

**Revised:** The depletion of TgANT leads to the lack of ATP in the ER, which may explain why the iTgANT strain has serious growth defects.

**Part I – Summary**

Please use this section to discuss strengths/weaknesses of study, novelty/significance, general execution and scholarship.

**Reviewer #1:** The manuscript has improved, with many important details that were lacking previously, as well as important data on PfANT, now included. It constitutes a significant and original contribution to the field.

**Reviewer #2:** The authors added a series of experiments to address the reviewers' comments and concerns. It is highly appreciated. The authors encountered a technical challenge in expressing ATP sensors in the parasite's ER. Hence, the evidence supporting the conclusion that TgANT1 transports ATP into ER is still missing, which is a key part of this manuscript. Are there any other strategies proving that the ATP content in the ER of WT is higher than that in the knockdown strain?

**Response：**We have also consulted other paper to find method for detecting the ATP content of the ER, but unfortunately, we found that the ATP sensor is currently the most commonly used method to detect the ATP content of the ER, and we have not found other better methods. We acknowledge that the accuracy of our results would be improved if we could detect the difference in the ER ATP content between iTgANT and wild-type strains.

**Reviewer #3:** As I stated on the original submission, the research and the findings are original and of significant importantce to the field. I had only a series of relatively minor issues and queries, all of which have been assiduously addressed.

As such, I now recommend acceptance of this manuscript.

**Part II – Major Issues: Key Experiments Required for Acceptance**

Please use this section to detail the key new experiments or modifications of existing experiments that should be absolutely required to validate study conclusions.

Generally, there should be no more than 3 such required experiments or major modifications for a "Major Revision" recommendation. If more than 3 experiments are necessary to validate the study conclusions, then you are encouraged to recommend "Reject".

**Reviewer #1:** My recommendations are relatively minor in nature and are outlined in Part III.

**Reviewer #2:** See the summary

**Reviewer #3:** none

**Part III – Minor Issues: Editorial and Data Presentation Modifications**
Please use this section for editorial suggestions as well as relatively minor modifications of existing data that would enhance clarity.

Reviewer #1: The localisation images have improved. However, all proteins used as markers (and their localisations) should be referred to in the relevant Figure legends (e.g. IMC and GAP45 in Fig. 1C, ALD in Fig. 4C).

**Response：**We are sorry for the lack of clarity in the description in the manuscript, we have made changes.

Fig 1 (C) TgANT-HA parasites were subjected to immunostaining using antibodies against the HA tag along with SERCA (sarco/endoplasmic reticulum Ca^2+^-ATPase, ER protein), The result shows that TgANT was complete co-localization with the ER-resident protein SERCA. But not co-localization with IMC (inner membrane complex, inner membrane protein) and GAP45 (gliding-associated protein 45, plasma membrane) of T. gondii. Scale bars: 2 μm.

Fig 4 **(C)** The Ty epitope at the C-terminal of TgANT was detected by IFA (red) at the appointed time after the 0.5 μg/ml ATc treatment. The results showed that TgANT was completely depletion after 24 hours of drug treatment. The cytoplasmic protein fructose-1,6-bisphosphate aldolase (ALD) of *T. gondii* as a marker.

There remains a need for editing, and for the following corrections/clarifications:

Line 56/57: “protein misleading” – should this be “protein misfolding”?
**Response：**Very sorry for the errors in our manuscript, we have revised it in the new manuscript, the correct description is “protein misfolding”

Line 89 – should specify what type of mammalian cells (HeLa?) are being referred to.
**Response：**We are sorry for the lack of clarity in the description in the manuscript, we have made changes. The mammalian cells are referred to HeLa cells.

“Unlike the severe phenotypic defects of *Arabidopsis thaliana* caused by ER-ANT1, the depletion of AXER did not cause severe growth damage to the HeLa cells”

Line 108 – “dense granules” should not be referred to as proteins.
**Response：**We have revised in our new manuscript.

“In the process of host-pathogen interactions, *T.gondii* utilizes a large amount of specialized secreted proteins to modify host cytokines to promote invasion and replication, such as microneme protein (MICs), rhoptry protein (ROPs), and **dense granules protein (GRAs)**”

Line 130 – what were the CRISPR phenotype value and TMD number set to?
**Response：**We believe that ER ATP is essential for the growth of *T. gondii*, so the ER ATP/ADP transporter may be an essential gene for *T. gondii*, and these essential genes of *T. gondii* generally have low CRISPR phenotype values. To narrow the screen scope, we set the phenotype value to less than -3. Besides, most transporter proteins have multiple transmembrane domains (TMD), also in order to narrow screen scope, we set the TMD number to be greater than 6. We initially envisaged screening target gene with this approach slowly, but we found the gene of interest on the first screen.

Line 175 – “time linear import of at least 20 min” – the uptake of ATP is not linear for this amount of time according to the graph.  **Response：**We have revised in our new manuscript. “The result showed ATP was imported into intact bacterial cells harboring TgANT and revealed a very high transport capacity for radioactive ATP”.

Line 185 – “Kinetics of” should be replaced with “Time course for”.
**Response：**We have revised in our new manuscript. “Fig 2B Time course for [α-^32^P] ATP uptake into intact *E. coli* cells.”

A number of Figure legends (e.g. Fig. 2, Fig. 6, Fig. 9, Fig. 10) now specify that three independent experiments were performed; however, it still needs to be stated what the data shown are (mean +/- SEM of the three independent experiments?)
**Response：**We are sorry for the lack of clarity in the description in the manuscript, we have made changes. Data are reported as the mean of at least three independent experiments and are shown with the standard error of the mean (SEM).

Line 206 – In this section, as well as in the Fig. 3A legend, it is important to state from where (the extracellular medium?), and at what time point, the presence of radioactivity is being tested for.
**Response：**We are sorry for the lack of clarity in the description in the manuscript, we have made changes.

**Fig3 Back exchange experiments with *E. coli* cells.**

**(A)** *E. coli* cells expressing TgANT were preloaded with 50nM radioactive [α-^32^P] ATP for 5 min, followed by carried out back exchange experiment with 5 μM non-labeled ATP, ADP, or PBS for 3 min and terminated by rapid centrifugation. Then, 2 μL sample of the supernatant were loaded onto the TLC PEI cellulose plate. The supernatant of the broken *E. coli* cells was used to detect the metabolic activity (lane 4). **(B)** and **(C)** *E. coli* cells harboring the plasmid encoding TgANT were incubated with 50 nM [α-^32^P] ATP for 10 min. Then, efflux was induced by the addition of fifty-fold unlabeled ATP **(B)** or ADP **(C)** for 1min, 2min, 5min and 8min. Means ± s.e.m., three independent assays.

Do the terms “disrupted [E. coli]” and “broken [E. coli]” mean lysed?
**Response：**Yes**,** to detect the metabolic activity of *E. coli*, the [α-^32^P] ATP preloaded *E. coli* were broken by ultrasound. Then, rapid centrifugation and the supernatant of the broken *E. coli* cells was used to carry out the TLC.

Line 253 – area relative to what (i.e. what are the plaque sizes being compared to)?
**Response：**The “relative area” in our manuscript is not the actual area of the plaque, we used the pixel size (calculated by Photoshop) instead of the actual area, all plaque photo were obtained under the same conditions. We have revised the pictures and legends.

**Fig4 Conditional knockdown of TgANT results in severe growth defects.**

**(E) and (F)** Parasites were grown ±0.5 μg/ml ATc for 7 days to form plaques on HFF monolayers **(E)**. plaque size presented as relative area (pixel size calculated by Photoshop) from three independent assays **(F)**. ***p ≤ 0.001, Student's t-test, three independent assays.

**Fig5 TgANT complementation fully restored the growth defects of TgANT depletion mutants.**

**(E) and (F)** Plaque assays comparing the growth of TgANT depletion strain and TgANT complementation strain. Parasites were grown ±0.5 μg/ml ATc for 7 days to form plaques on HFF monolayers **(E)**. plaque size presented as relative area (pixel size calculated by Photoshop) from three independent assays **(F)** ***p ≤ 0.001, Student's t-test, three independent repeated.

Would the data in Fig. 5G be better placed in Fig. 4, since they relate to the growth phenotype of TgANT knockdown parasites, and not to TgANT complementation?
**Response：**This is a very constructive comment and we have revised it in our new manuscript. The data in Fig. 5G have been placed in Fig. 4 (Fig. 4G) and the associated statement and legend have been revised in the new manuscript.

Given that the authors have not investigated the kinetics of TgANT or PfANT or their substrate affinities, they should not refer to the “affinity” for substrates (as they do in lines 425, 427, 604, 605). It would be more accurate to refer to the degree of inhibition caused by the various non-radiolabelled molecules at the concentration tested.

**Response：**
This is a very constructive comment and we have revised it in our new manuscript

“As expected, PfUGT also revealed a transport capacity for ATP and ADP (Fig 9B).”

“The ability of *E. coli* to take up radioactive ATP was inhibited in the presence of non-radioactive CTP, but the transport capacity for CTP lower than ATP and ADP (Table. 2)”

“Competition experiments showed that PfANT also has a certain transport capacity for GNF179, and its presence can inhibit the uptake of [α-^32^P] ATP.”

“Interestingly, PfANT not only able to transport ATP and ADP, but also has a certain ability to transport CTP and the antimalarial drug GNF179.”

Lines 575 – 579: the information in these lines that does not come from this study should be accompanied by references.

**Response：**We have added references to the new manuscript.

Line 589: “the ER stress caused by ER ATP/ADP transporter” – should this be "the ER stress caused by a deficiency of the ER ATP/ADP transporter"?

**Response：**This is a very important suggestion and we have revised it in our new manuscript

“However, the ER stress caused by a deficiency of the ER ATP/ADP transporter does not seem to be universal. Mutant plants lacking ER-ANT1 exhibit a severe dwarf phenotype but do not cause apparent ER stress in *Arabidopsis thaliana*.”

Reviewer #2: None

Reviewer #3: none
