# Supplementary material for: A new adenine nucleotide transporter located in the ER is essential for maintaining the growth of Toxoplasma gondii
Source: PLoS Pathog. 2022 Jul 5;18(7):e1010665. doi: 10.1371/journal.ppat.1010665 (PMC9286291; doi:10.1371/journal.ppat.1010665)
Supplement: Attachment — Submitted filename: Response to Reviewers.docx [file ppat.1010665.s010.docx]

**Reviewer #1:** The last sentence of the discussion still makes it sound like ATP levels were measured and found to be lacking in the ER: "The depletion of TgANT leads to the lack of ATP in the ER, which may explain why the iTgANT strain has serious growth defects."

Maybe this sentence could be modified to something like: "The depletion of TgANT likely leads to a lack of ATP in the ER, which may explain why the iTgANT strain has serious growth defects."

**Response：**We have revised it in our new manuscript.

**Before:** The depletion of TgANT leads to the lack of ATP in the ER, which may explain why the iTgANT strain has serious growth defects.

**Revised:** The depletion of TgANT **likely** leads to a lack of ATP in the ER, which may explain why the iTgANT strain has serious growth defects.
